# The Comparative Anatomy and Physiology of the Salivary Glands in Man and Other Vertebrate Animals

**Published:** 1853-03

**Authors:** C. L. Bernard


*The Comparative Anatomy and Physiology of the Salivary Glands in Man and other Vertebrate Animals.* By M. C. L. Bernard.—Anatomy shows the group of salivary glands as an homogeneous apparatus, of which the different parts are identical in structure. Physiological analysis, on the other hand, teaches that the secreted products are of different composition. In spite of the mixture of the different kinds of saliva in the mouth, their functions remain distinct. The characteristic of the parotid is to secrete for mastication; that of the submaxillary for taste; that of the sublingual and buccal for deglutition. We cannot expect to find in birds the homogeneous types of the parotid and submaxillary glands, because the functions connected with these two organs, namely, mastication and gustation (taste,) are wanting. The viscid and glairy secretion which flows from their glands has nothing in common with the parotid and submaxillary fluids of mammalia, but it is identical with that furnished by the sublingual and buccal glands. In mammalia, which masticate hard and dry articles of food, the parotid attains its extreme development; in those which, as the phoca, live in water, and feed off moist substances, this gland is atrophied, or absent altogether. The other glands retain their size according to the necessity of the functions over which they preside.—*L' Union Medicale,* 1852.

